# Chemoradiotherapy Is Superior to Radiotherapy Alone in Esophageal Cancer Patients Older Than 65 Years: A Propensity Score-Matched Analysis of the SEER Database

**DOI:** 10.3389/fonc.2021.736448

**Published:** 2021-09-07

**Authors:** Xiaojie Xia, Qing Gao, Xiaolin Ge, Zeyuan Liu, Xiaoke Di, Xinchen Sun, Yan Yang

**Affiliations:** ^1^Department of Radiation Oncology, Jiangsu Province Hospital, Nanjing, China; ^2^Department of Radiation Oncology, Nanjing Medical University First Affiliated Hospital, Nanjing, China; ^3^The First School of Clinical Medicine, Nanjing Medical University, Nanjing, China

**Keywords:** esophageal cancer, SEER, elderly patients, chemoradiation, survival analysis

## Abstract

**Introduction:**

Radiotherapy (RT) is the main treatment for unoperated esophageal cancer (EC) patients. It is controversial whether adding chemotherapy (CT) to RT is beneficial for elderly EC patients. The purpose of our study was to compare the efficacy of chemoradiotherapy (CRT) with RT alone for non-surgical elderly esophageal cancer patients.

**Methods:**

A total of 7,101 eligible EC patients older than 65 years diagnosed between 2000 and 2018 were collected from the Surveillance, Epidemiology, and End Results (SEER) database. All the samples were divided into the radiotherapy group and the chemoradiotherapy group. After being matched by propensity score matching (PSM) at a 1:1 ratio, 3,020 patients were included in our analysis. The Kaplan–Meier method and log-rank test were applied to compare overall survival (OS) and cancer-specific survival (CSS).

**Results:**

After PSM, the clinical characteristics of patients between the RT and CRT groups were comparable. For EC patients older than 65 years, the 3-year OS and CSS in the CRT group were 21.8% and 27.4%, and the 5-year OS and CSS in the CRT group were 12.7% and 19.8%, respectively. The 3-year OS and CSS in the RT group were 6.4% and 10.4%, and the 5-year OS and CSS in the RT group were 3.5% and 7.2%, respectively. Next, these patients were divided into five subgroups based on the age stratification (ages 65–69; 70–74; 75–79; 80–84; ≥85). In each subgroup analysis, the 3- and 5-year OS and CSS showed significant benefits in the CRT group rather than in the RT group (all p < 0.05). We were unable to assess toxicities between the two groups due to a lack of correlated information.

**Conclusions:**

CRT could improve OS and CSS for non-surgical EC patients older than 65 years. Adding chemotherapy to radiation showed a significant prognostic advantage for elderly esophageal cancer patients.

## Introduction

Esophageal cancer is the second tumor of the digestive system besides gastric cancer (1). Esophageal squamous cell carcinoma (ESCC) is the major histology reported in Asian countries; adenocarcinoma in Western countries (2). Even though we have made great progress in esophageal cancer treatment, the prognosis of locally advanced tumors is still poor with a 5-year survival rate ranging from 15% to 25% (3). Most patients with esophageal cancer are not diagnosed until the disease is advanced, and only about 20% of the advanced esophageal cancers can be surgically removed (4). Esophagectomy remains the main treatment for esophageal cancer, but the overall 5-year survival rate with surgery alone is estimated at 16%–33% (5). Definite chemoradiotherapy (CRT) is a satisfactory treatment for esophageal cancer patients who are rejected or not suitable for surgery. JCOG9906, a phase II study of CRT for stage II/III ESCC, showed a promising outcome with a 5-year survival rate of 36.8% (6). Even for stage IV patients, chemoradiotherapy was an effective palliative treatment (7).

In the USA, 44% of EC patients are over 60 years. Moreover, approximately 69.8% of EC patients in males of China are older than 60 years (8, 9). Global aging and improved life expectancy indicate that cancer in older patients is becoming an increasingly common social issue. Many elderly patients are not candidates for surgical resection due to medical comorbidities and poorer physiologic status (10). Radical radiation therapy is usually their primary treatment. Because the side effects of radiotherapy combined with chemotherapy are more serious than radiotherapy alone (11), whether radiotherapy combined with chemotherapy is a better choice for elderly esophageal cancer patients has not yet been unified. We thus evaluated the effectiveness of CRT compared with RT alone in EC patients older than 65 years based on a propensity score-matched (PSM) analysis of the SEER database. Furthermore, we performed age-stratified analyses to explore survival differences between the two groups, respectively.

## Methods

### Study Population

The Surveillance, Epidemiology, and End Results (SEER) database (http://seer.cancer.gov/) includes clinical information on about 30% of cancer patients in the USA. We enrolled patients aged 65 or older and diagnosed with EC between January 2000 and December 2018. The following are the exclusion criteria: (I) cases that did not receive radiotherapy or receive other radiotherapy methods other than beam radiation; (II) surgical or unknown cases; (III) without AJCC 6th staging; (IV) the pathological type is non-squamous cell carcinoma or adenocarcinoma; (V) cases where the location of the tumor is unknown; and (VI) cases with absence or zero survival time. Information was extracted for age, sex, ethnicity, tumor size, pathological type, stage, radiotherapy, chemotherapy, and month of survival.

### Statistical Analysis

We used Pearson chi-square analysis to determine variables between the two groups. To balance the covariance and reduce the bias of efficacy evaluation, we performed a 1:1 propensity score matching between the chemoradiotherapy group and the radiotherapy group alone. Then, we divided these patients into five subgroups based on the age stratification (ages 65–69; 70–74; 75–79; 80–84; ≥85). The Kaplan–Meier method and log-rank test were used to compare the overall survival (OS) and cancer-specific survival (CSS) of the two groups in IBM SPSS Statistics for Windows, Version 26.0 (IBM, Armonk, NY, USA); p < 0.05 indicated that the difference was statistically significant.

## Results

### Patients’ Survival

**Figure 1:**
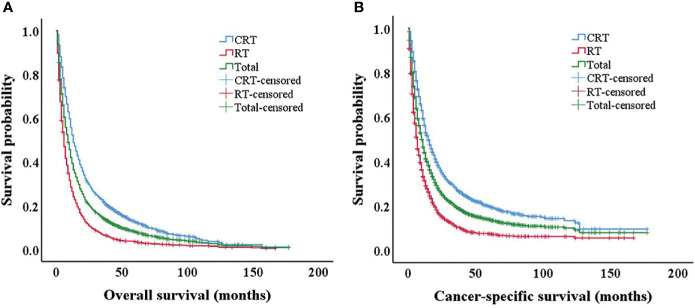
Kaplan–Meier survival analysis after propensity score matching (PSM). Overall survival **(A)** and cancer-specific survival **(B)** of all patients after PSM. PSM, propensity score matching.

A total of 44,373 EC patients older than 65 years diagnosed between 2000 and 2018 of the SEER database were initially enrolled, and 37,272 patients were excluded based on exclusion criteria. All characteristics between the RT and CRT groups in the 7,101 eligible patients are shown in **Table S1**. After six characteristics being matched by PSM at a 1:1 ratio, 3,020 patients were included in our analysis (**Table 1**). AS shown in **Figure 1**, The 3- and 5-year OS of the whole patients were 14.1% and 8.1%, respectively. The 3- and 5-year CSS of the whole group were 19.2% and 13.8%, respectively. The median OS and CSS of the whole group were 9 and 10 months, respectively. After propensity score matching (PSM), there were 1,510 patients in the radiotherapy (RT) alone group and the radiochemotherapy (CRT) group, respectively. In the RT alone group, the 3- and 5-year OS were 6.4% and 3.5%, and the 3- and 5-year CSS were 10.4% and 7.2%, respectively. In the CRT group, the 3- and 5-year OS and CSS rates were 21.8% and 12.7% and 27.4% and 19.8%, respectively. In EC patients older than 65 years, the survival rate was significantly better in the CRT group than in the RT alone group.

**Table 1:** Comparison of baseline variables between radiotherapy and chemoradiotherapy groups in the original and matched datasets in cases of metastatic esophageal cancer.

Variables	Original data set			Matched data set		
	CRT	RT	p	CRT	RT	p
Total	5561	1540		1510	1510	
Age			<0.001			<0.001
65–69	1551	223		178	223	
70–74	1391	243		253	243	
75–79	1319	327		298	327	
80–84	874	353		490	353	
85+	426	394		291	364	
Gender			<0.001			0.936
Female	1344	460		437	435	
Male	4217	1080		1073	1075	
Race			0.102			0.017
White	4715	1267		1299	1244	
Black	535	172		124	167	
Other^*^	306	100		84	98	
Unknown	5	1		3	1	
Tumor location			0.251			0.66
Upper	661	183		206	176	
Middle	1387	419		399	408	
Lower	3249	875		865	864	
Overlapping	264	63		40	62	
Pathological type			0.719			0.798
EAC	2997	822		810	817	
ESCC	2564	718		700	693	
AJCC Stage 6th			<0.001			<0.001
I	853	340		140	340	
IIA	1113	247		458	245	
IIB	623	122		150	122	
III	1655	288		449	277	
IVA	207	41		65	38	
IVB	1110	502		248	488	

*Asian, Pacific Islander, and American Indian/Alaskan Native.

RT, radiotherapy; CRT, chemoradiotherapy; ESCC, esophageal squamous cell carcinoma; EAC, esophageal adenocarcinoma.

### Patients’ Characteristics

After PSM, 3,020 well-balanced elderly EC patients were available in the RT alone group and CRT group (1,510 patients in each group). The median age was 75 years (range 65–99 years), with 2,148 (71.1%) being male. The clinical-stage distribution was as follows: stage I 480 (15.89%), stage II A 703 (23.28%), stage II B 272 (9.01%), stage III 726 (24.04%), stage IV A 103 (3.41%), and stage IV B 736 (24.37%). More than half of the tumors were located in the lower esophagus (57.3%). There are 1,393 patients with esophageal squamous cell carcinoma and 1,627 patients with adenocarcinoma. The six clinicopathological variables were well balanced in 3,020 patients. Details are summarized in **Table 1**.

### Survival Analysis

**Figure 2:**
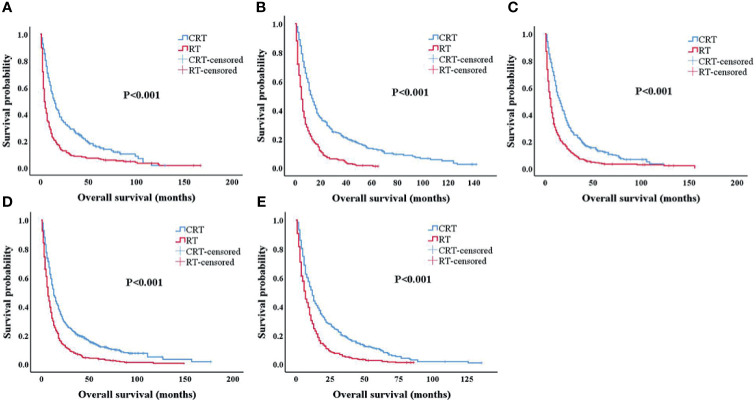
Kaplan–Meier survival analysis after propensity score matching (PSM). Overall survival for patients 65–69 years old **(A)**, 70–74 years old **(B)**, 75–79 years old **(C)**, 80–84 years old **(D)**, and 85+ years old **(E)** after PSM. PSM, propensity score matching.

In order to compare the survival rate of the CRT and RT-alone groups at different ages, we divided 3,020 patients into five age stages (ages 65–69; 70–74; 75–79; 80–84; ≥85, respectively). There were 401, 496, 625, 843, and 655 patients in ages 65–69; 70–74; 75–79; 80–84; and ≥85, respectively. In patients treated with chemoradiotherapy, the 3-year OS rates for patients with ages 65–69; 70–74; 75–79; 80–84; and ≥85 were 26.6%, 22.4%, 22.0%, 21.1%, and 19.3%, respectively. The 5-year OS rates for patients with ages 65–69; 70–74; 75–79; 80–84; and ≥85 were 14.7%, 12.9%, 12.3%, 12.6%, and 10.2%, which were clearly better than patients treated with radiotherapy alone (p < 0.001) (**Figure 2**).

**Figure 3:**
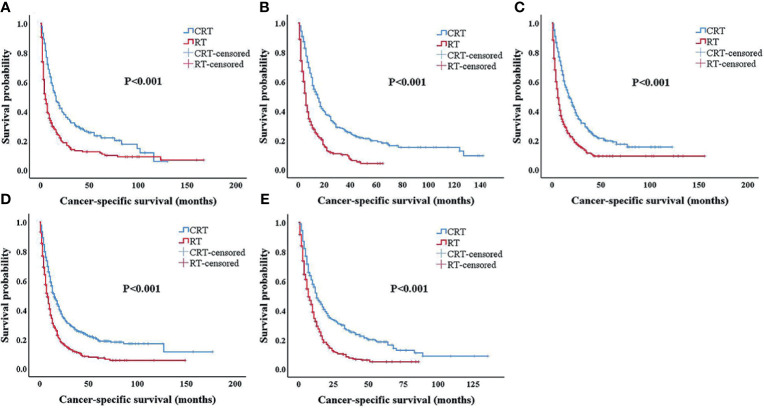
Kaplan–Meier survival analysis after propensity score matching (PSM). Cancer-specific survival for patients 65–69 years old **(A)**, 70–74 years old **(B)**, 75–79 years old **(C)**, 80–84 years old **(D)**, and 85+ years old **(E)** after PSM. PSM, propensity score matching.

In patients treated with radiotherapy alone, the 3-year CSS rates for patients with ages 65–70; 70–75; 75–79; 80–84; and ≥85 were 13.2%, 9.4%, 11.3%, 11.4%, and 7.4%, respectively; the 5-year CSS rates for patients with ages 65–69; 70–74; 75–79; 80–84; and ≥85 were 11.6%, 4.2%, 9.2%,7.2%, and 4.9%, respectively, which were clearly lower than in patients treated with chemoradiotherapy (**Figure 3**). The OS and CSS rates in patients treated with chemoradiotherapy of each age group were significantly higher than those in the RT-alone group (all p < 0.001).

## Discussion

ESCC is the leading type worldwide due to the highest rates occurring in Asia. Esophageal adenocarcinoma (EAC) is more popular than ESCC in the Western world in the last half-century (12). The morbidity and mortality of esophageal cancer in developing countries account for more than 80% worldwide (13), and the new cases in China account for about 50% of the world (14, 15). The incidence of esophageal cancer rose rapidly after 45 years. According to China’s estimated data in 2015, patients over the age of 75 accounted for about 20% in the distribution of EC morbidity and also had upper mortality by 31.1% (8). With the development of aging, cancer in older adults is turning into an increasingly common social problem. However, there is no consensus on the treatment of esophageal cancer in the elderly.

Patients over 70 years have poorer tolerance in esophagectomy than younger patients. Multiple retrospective analyses (16, 17) found that EC patients over 70 years old have significantly increased postoperative complications and perioperative mortality. Clinically, definite chemoradiotherapy is the standard therapy for non-surgical esophageal cancer patients. However, some elderly patients refused chemotherapy and received radiotherapy alone due to treatment complications or economic or psychological reasons. Is chemoradiotherapy better than radiotherapy alone for elderly patients? There are few random studies or bulk data case analysis targeting elderly patients with esophageal cancer at present.

In our study, we included a total of 7,101 EC patients over 65 years old diagnosed between 2000 and 2018 in the SEER database. The results of this research indicated that chemoradiotherapy can be used successfully to treat patients older than 65 years. After PSM, 3,020 patients were included for further analysis, the 3- and 5-year OS and CSS in the CRT group were longer than those in the RT-alone group in five subgroup analyses stratified by age (ages 65–69; 70–74; 75–79; 80–84; ≥85, respectively). The 5-year survival rate of elderly cancer patients undergoing radical radiotherapy or radiochemotherapy is 9.7%~30.1% (18, 19). Many previous clinical research results were consistent with our reports. Smith et al. (20) analyzed 2,626 EC patients older than 65 years from 1992 to 2002 in the SEER database; the 5-year survival rates of 623 patients (24%) that received radiotherapy and 1,024 patients (39%) received radiochemotherapy were 2% and 11%, respectively (p < 0.001). Xu et al. found that EC patients over 70 years old exhibited improved survival rates with CRT compared with RT alone (21). The OS and PFS in the CRT group versus the RT group were 17 versus 8 months and 14 versus 5 months (p = 0.01), respectively. Some previous reports had different results from our research as elderly EC patients get older and older. The results of the study by Zhang et al. revealed that survival was significantly better in the CRT group than in the RT-alone group for patients less than 72 years old. However, in terms of survival benefit for patients older than 72 years, CRT was not superior to RT alone (22). Jingu et al. reported that adding chemotherapy to radiotherapy for esophageal cancer in patients aged 80 years or older did not have significant OS benefit over radiotherapy alone (23). The simultaneously recent article which analyzed a total of 358 patients aged 80 years or older in the Japanese Nationwide Cancer Database showed that chemoradiotherapy for EC patients aged 80 years or older was a significant favorable prognostic factor for OS (24). Our research results were based on the SEER database, and we used PSM to balance the clinical features between the CRT and RT-alone groups. The results of our analysis were reliable due to a large number of patients in each age-stratified subgroup, ranging from 401 to 843. Fortunately, a two-arm, open, randomized multicenter Phase III trial with EC patients over 70 years old is going to explore the best treatment option for the elderly (25). Our results indicated that adding chemotherapy to radiotherapy did have therapeutic advantages regardless of how old the EC patients are. Numerous factors, including the radiation field and chemotherapy regimen, influence the curative effect of radiochemotherapy for elderly EC patients. Jing et al. enrolled a total of 137 elderly patients older than 70 years; 54 patients (39.4%) were allocated to the elective nodal irradiation (ENI) group and 83 patients (60.6%) to the involved field irradiation (IFI) group. The results showed that IFI resulted in decreased irradiation toxicities without sacrificing OS in elderly EC patients (26). A study including 184 EC patients aged ≥70 years who received oral single-agent CCRT (sCCRT) or double-agent CCRT (dCCRT) or RT alone at a single institution in China demonstrated that CCRT had significant survival benefits compared to RT alone, especially in the oral single-agent group (11). We need prospective clinical trials to search for the best combination of radiotherapy and chemotherapy.

In summary, our study findings suggested that adding chemotherapy to radiation for elderly patients with esophageal cancer should be implemented as much as possible, even in patients aged 80 years or older. Furthermore, we need to find the optimal combination of radiotherapy and chemotherapy to maximize the survival benefit of elderly EC patients. Further prospective studies are warranted to verify our results.

## Data Availability Statement

The original contributions presented in the study are included in the article/**Supplementary Material**. Further inquiries can be directed to the corresponding authors.

## Author Contributions

Study concept and design: XX and YY. Data acquisition and data analysis: QG. Draft and revision of important content: XX and XD. Final draft approval: XS and XG. Literature research: ZL and XD. All authors contributed to the article and approved the submitted version.

## Conflict of Interest

The authors declare that the research was conducted in the absence of any commercial or financial relationships that could be construed as a potential conflict of interest.

## Publisher’s Note

All claims expressed in this article are solely those of the authors and do not necessarily represent those of their affiliated organizations, or those of the publisher, the editors and the reviewers. Any product that may be evaluated in this article, or claim that may be made by its manufacturer, is not guaranteed or endorsed by the publisher.

## References

[1] Yennurajalingam S Kang JH Cheng HY Chisholm GB Kwon JH Palla SL. Characteristics of Advanced Cancer Patients With Cancer-Related Fatigue Enrolled in Clinical Trials and Patients Referred to Outpatient Palliative Care Clinics. J Pain Symptom Manage (2013) 45(3):534–41. 10.1016/j.jpainsymman.2012.02.013 PMC3855412 22917716

[2] Short MW Burgers KG Fry VT. Esophageal Cancer. Am Fam Physician (2017) 95(1):22–8. 28075104

[3] Pennathur A Gibson MK Jobe BA Luketich JD. Oesophageal Carcinoma. Lancet (2013) 381(9864):400–12. 10.1016/s0140-6736(12)60643-6 23374478

[4] Kim DE Kim UJ Choi WY Kim MY Kim SH Kim MJ. Clinical Prognostic Factors for Locally Advanced Esophageal Squamous Carcinoma Treated After Definitive Chemoradiotherapy. Cancer Res Treat (2013) 45(4):276–84. 10.4143/crt.2013.45.4.276 PMC3893325 24454000

[5] Tepper J Krasna MJ Niedzwiecki D Hollis D Reed CE Goldberg R. Phase III Trial of Trimodality Therapy With Cisplatin, Fluorouracil, Radiotherapy, and Surgery Compared With Surgery Alone for Esophageal Cancer: CALGB 9781. J Clin Oncol (2008) 26(7):1086–92. 10.1200/jco.2007.12.9593 PMC5126644 18309943

[6] Kato K Muro K Minashi K Ohtsu A Ishikura S Boku N. Phase II Study of Chemoradiotherapy With 5-Fluorouracil and Cisplatin for Stage II-III Esophageal Squamous Cell Carcinoma: JCOG Trial (JCOG 9906). Int J Radiat Oncol Biol Phys (2011) 81(3):684–90. 10.1016/j.ijrobp.2010.06.033 20932658

[7] Jingu K Umezawa R Matsushita H Sugawara T Kubozono M Yamamoto T. Chemoradiotherapy for T4 and/or M1 Lymph Node Esophageal Cancer: Experience Since 2000 at a High-Volume Center in Japan. Int J Clin Oncol (2016) 21(2):276–82. 10.1007/s10147-015-0896-2 26324841

[8] Chen W Zheng R Baade PD Zhang S Zeng H Bray F. Cancer Statistics in China, 2015. CA Cancer J Clin (2016) 66(2):115–32. 10.3322/caac.21338 26808342

[9] Trivers KF Sabatino SA Stewart SL. Trends in Esophageal Cancer Incidence by Histology, United States, 1998-2003. Int J Cancer (2008) 123(6):1422–8. 10.1002/ijc.23691 18546259

[10] Won E Ilson DH. Management of Localized Esophageal Cancer in the Older Patient. Oncologist (2014) 19(4):367–74. 10.1634/theoncologist.2013-0178 PMC3983810 24664485

[11] Zhao L Zhou Y Pan H Yin Y Chai G Mu Y. Radiotherapy Alone or Concurrent Chemoradiation for Esophageal Squamous Cell Carcinoma in Elderly Patients. J Cancer (2017) 8(16):3242–50. 10.7150/jca.20835 PMC5665040 29158796

[12] He H Chen N Hou Y Wang Z Zhang Y Zhang G. Trends in the Incidence and Survival of Patients With Esophageal Cancer: A SEER Database Analysis. Thorac Cancer (2020) 11(5):1121–8. 10.1111/1759-7714.13311 PMC7180574 32154652

[13] Torre LA Bray F Siegel RL Ferlay J Lortet-Tieulent J Jemal A. Global Cancer Statistics, 2012. CA Cancer J Clin (2015) 65(2):87–108. 10.3322/caac.21262 25651787

[14] Yao J. Status of Radiotherapy for Unoperated Esophageal Cancer. China Foreign Med Treat (2017) 16:195–8. 10.16662/j.cnki.1674-0742.2017.16.195

[15] Chen Y Guo L Cheng X Wang J Zhang Y Wang Y. With or Without Consolidation Chemotherapy Using Cisplatin/5-FU After Concurrent Chemoradiotherapy in Stage II-III Squamous Cell Carcinoma of the Esophagus: A Propensity Score-Matched Analysis. Radiother Oncol (2018) 129(1):154–60. 10.1016/j.radonc.2017.10.031 29122362

[16] Tapias LF Muniappan A Wright CD Gaissert HA Wain JC Morse CR. Short and Long-Term Outcomes After Esophagectomy for Cancer in Elderly Patients. Ann Thorac Surg (2013) 95(5):1741–8. 10.1016/j.athoracsur.2013.01.084 PMC3732120 23500043

[17] Jing W Guo H Kong L Zhang Y Wang H An C. Clinical Outcomes of Elderly Patients (≥70 Years) With Resectable Esophageal Squamous Cell Carcinoma Who Underwent Esophagectomy or Chemoradiotherapy: A Retrospective Analysis From a Single Cancer Institute. Med (Baltimore) (2016) 95(50):e5630. 10.1097/md.0000000000005630 PMC5268052 27977606

[18] Li X Zhao LJ Liu NB Zhang WC Pang QS Wang P. Feasibility and Efficacy of Concurrent Chemoradiotherapy in Elderly Patients With Esophageal Squamous Cell Carcinoma: A Respective Study of 116 Cases From a Single Institution. Asian Pac J Cancer Prev (2015) 16(4):1463–9. 10.7314/apjcp.2015.16.4.1463 25743816

[19] Chen M Liu X Han C Wang X Zhao Y Pang Q. Does Chemoradiotherapy Benefit Elderly Patients With Esophageal Squamous Cell Cancer? A Propensity-Score Matched Analysis on Multicenter Data (3JECROG R-03a). BMC Cancer (2020) 20(1):36. 10.1186/s12885-019-6461-z 31941487 PMC6964023

[20] Smith GL Smith BD Buchholz TA Liao Z Jeter M Swisher SG. Patterns of Care and Locoregional Treatment Outcomes in Older Esophageal Cancer Patients: The SEER-Medicare Cohort. Int J Radiat Oncol Biol Phys (2009) 74(2):482–9. 10.1016/j.ijrobp.2008.08.046 19289262

[21] Xu HY Du ZD Zhou L Yu M Ding ZY Lu Y. Safety and Efficacy of Radiation and Chemoradiation in Patients Over 70 Years Old With Inoperable Esophageal Squamous Cell Carcinoma. Oncol Lett (2014) 7(1):260–6. 10.3892/ol.2013.1694 PMC3861579 24348860

[22] Zhang P Xi M Zhao L Shen JX Li QQ He LR. Is There a Benefit in Receiving Concurrent Chemoradiotherapy for Elderly Patients With Inoperable Thoracic Esophageal Squamous Cell Carcinoma? PloS One (2014) 9(8):e105270. 10.1371/journal.pone.0105270 25133495 PMC4136816

[23] Jingu K Takahashi N Murakami Y Ishikawa K Itasaka S Takahashi T. Is Concurrent Chemotherapy With Radiotherapy for Esophageal Cancer Beneficial in Patients Aged 80 Years or Older? Anticancer Res (2019) 39(8):4279–83. 10.21873/anticanres.13592 31366518

[24] Jingu K Numasaki H Toh Y Nemoto K Uno T Doki Y. Chemoradiotherapy and Radiotherapy Alone in Patients With Esophageal Cancer Aged 80 Years or Older Based on the Comprehensive Registry of Esophageal Cancer in Japan. Esophagus (2020) 17(3):223–9. 10.1007/s10388-020-00725-w 32088786

[25] Li C Wang X Wang X Han C Wang P Pang Q. A Multicenter Phase III Study Comparing Simultaneous Integrated Boost (SIB) Radiotherapy Concurrent and Consolidated With S-1 Versus SIB Alone in Elderly Patients With Esophageal and Esophagogastric Cancer - the 3JECROG P-01 Study Protocol. BMC Cancer (2019) 19(1):397. 10.1186/s12885-019-5544-1 31036088 PMC6489222

[26] Jing W Zhu H Guo H Zhang Y Shi F Han A. Feasibility of Elective Nodal Irradiation (ENI) and Involved Field Irradiation (IFI) in Radiotherapy for the Elderly Patients (Aged ≥ 70 Years) With Esophageal Squamous Cell Cancer: A Retrospective Analysis From a Single Institute. PloS One (2015) 10(12):e0143007. 10.1371/journal.pone.0143007 26636574 PMC4670202

